# Direct growth of few-layer graphene on AlN-based resonators for high-sensitivity gravimetric biosensors

**DOI:** 10.3762/bjnano.10.98

**Published:** 2019-04-29

**Authors:** Jimena Olivares, Teona Mirea, Lorena Gordillo-Dagallier, Bruno Marco, José Miguel Escolano, Marta Clement, Enrique Iborra

**Affiliations:** 1 GMME-CEMDATIC, ETSI de Telecomunicación, Universidad Politécnica de Madrid, Spain

**Keywords:** biomolecule detection, graphene integration, gravimetric biosensor, surface functionalization

## Abstract

We present the successful growth of few-layer graphene on top of AlN-based solidly mounted resonators (SMR) using a low-temperature chemical vapour deposition (CVD) process assisted by Ni catalysts, and its effective bio-functionalization with antibodies. The SMRs are manufactured on top of fully insulating AlN/SiO_2_ acoustic mirrors able to withstand the temperatures reached during the CVD growth of graphene (up to 650 °C). The active AlN films, purposely grown with the *c*-axis tilted, effectively excite shear modes displaying excellent in-liquid performance, with electromechanical coupling and quality factors of around 3% and 150, respectively, which barely vary after graphene integration. Raman spectra reveal that the as-grown graphene is composed of less than five weakly coupled layers with a low density of defects. Two functionalization protocols of the graphene are proposed. The first one, based on a covalent binding approach, starts with a low-damage O_2_ plasma treatment that introduces a controlled density of defects in graphene, including carboxylic groups. After that, 1-ethyl-3-(3-dimethylaminopropyl)carbodiimide hydrochloride/*N*-hydroxysuccinimide (EDC/NHS) chemistry is used to covalently bind streptavidin molecules to the surface of the sensors. The second functionalization protocol is based on the non-covalent bonding of streptavidin on hydrophobic graphene surfaces. The two protocols end with the effective bonding of biotinylated anti-IgG antibodies to the streptavidin, which leaves the surface of the devices ready for possible IgG detection.

## Introduction

Gravimetric biosensors based on microscale mechanical or electromechanical resonators have attracted significant interest in recent years mainly due to the high sensitivity and selectivity they can attain if properly functionalized [1–2], the small sample volumes they require for operation when combined with microfluidics [3], their high-speed response and label-free operation [4]. Piezoelectric resonators based on thin films of, e.g., AlN or ZnO offer significantly greater sensitivities than conventional quartz crystal microbalances (QCMs), and can be configured in sensor arrays and integrated (on-wafer or in-package) along with their driving electronics and microfluidic arrangements, offering compact and inexpensive measurement systems [5–6]. Among the wide variety of thin-film resonators exploiting different acoustic modes such as surface acoustic waves (SAW), Lamb waves or bulk acoustic waves (BAW), the latter appear to be the preferred choice on account of the high operation frequencies they can achieve upon reducing the thickness of the piezoelectric active films. Particularly, solidly mounted resonators (SMR), providing acoustic isolation through acoustic reflectors instead of air cavities, are well suited for in-liquid operation and microfluidics integration [3].

A critical step in the manufacturing of gravimetric biosensors is the functionalization of their active surface, which provides the sensor with the desired selectivity and sensitivity to the targeted species. Selectivity mainly depends on the specificity of the receptor (e.g., for proteins, aptamers or antibodies) to the targeted species and the non-specific binding degree of other species that can be achieved; effective functionalization platforms should accommodate a large density of adequately oriented receptors and be easily passivated against non-specific binding. Sensitivity in acoustic resonators depends on their ability to detect small changes of the resonant frequency, which is essentially a matter of design. The functionalization schemes also play an essential role. In fact, for a given design, the sensitivity not only depends on the density of the attached active receptors, but also on their distance to the device surface. For example, the interaction length of shear-mode resonators operating in liquid appears to be limited to the near-surface region [7]. A decrease of the sensitivity to the added mass has been observed when the surface-receptor cross linkers length increases [8], which makes zero-length cross linkers or direct bonding schemes attractive.

Functionalization platforms are typically based on solid or porous gold films [9], owing to their outstanding properties in terms of electrical conductivity combined with their chemical stability and their ability to alter their chemistry under controlled conditions. Recently, functionalized graphene and graphene oxide have attracted the attention of the scientific community due to their extraordinary prospects for novel applications, such as highly sensitive biosensors that may offer continuous label-free measurement of key bio-active cell molecules [10]. Few-layer graphene grown on top of gravimetric transducers offers, a priori, one of the most versatile functionalization platforms. On one hand, graphene containing defects (COOH groups) can be covalently functionalized by using an EDC/NHS zero-cross linker, which allows for the binding of primary amines present in proteins and antibodies [11–12]. On the other hand, defect-free graphene is highly hydrophobic, and promotes (like carbon nanotubes [13]) the direct non-covalent binding of molecules like streptavidin, which is the basis of the functionalization scheme based on biotinylated receptors. Both methods result in short chains from the surface to the receptor, which optimizes the interaction of the acoustic field near the device surface with the targeted species, thus allowing for a more sensitive detection.

So far, only few works dealing with the integration of graphene-based functionalization platforms on piezoelectric resonators have been reported. Besides, high-quality graphene-based layers are typically transferred to the active area of the devices after being grown at high temperatures on copper catalysts [14], or from liquid suspensions [15], but never directly grown on top of the resonators. However, not all the applications require high-quality single-layer graphene; in fact, few-layer graphene appears to be more appropriate for the covalent functionalization scheme based on the generation of defects (COOH groups) through a plasma treatment.

In this work, we demonstrate that few-layer graphene can be grown directly on top of SMR-type acoustic resonators without a noticeable degradation of their characteristics and then functionalized to manufacture gravimetric biosensors, which eliminates the need to use complex transfer methods. Defect-free few-layer graphene was selectively grown through a low-temperature (650 °C) CVD process on Ni [16] thin-film catalysts previously evaporated and patterned over the top electrode of the shear-mode SMRs. The active area of the resonators was subsequently functionalized using both covalent and non-covalent schemes that ended with the successful detection of antibodies without significant worsening of the resonator performance. Opposite to graphene transfer methods, all the technological processes proposed to achieve the final device are compatible with conventional microfabrication technologies, which offers an easy and inexpensive method for the mass production of biosensors.

## Results and Discussion

### Characterization of graphene grown on nickel catalyst layers

**Figure 1:**
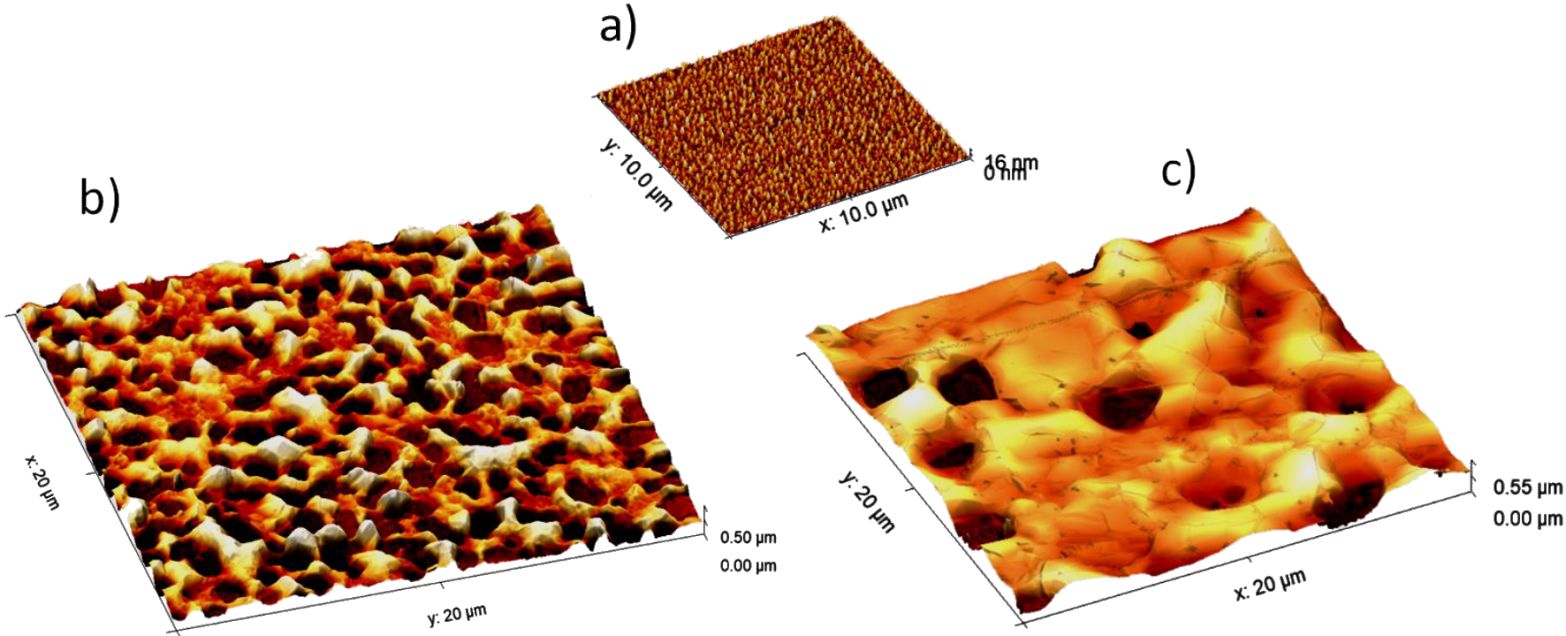
3D AFM image of the surface of the Ni film: a) as grown, b) after graphene growth with a high heating rate (200 °C/min) and c) after graphene growth with a slow heating rate (50 °C/min). Observe the folded structure of the graphene on the Ni surface in c).

The thickness of the catalyst Ni layers was reduced to 100 nm, which was thin enough as not to compromise the resonator performance but sufficient to avoid the formation of Ni droplets on the surface of the SMRs during the heating processes due to dewetting [17]. However, the surface of the Ni film suffered from restructuration during the CVD process that strongly depended on the heating rate. In order to assess the surface of the films after the growth of graphene, the samples were analysed by atomic force microscopy (AFM) using a Molecular Imaging Pico LE apparatus operated in contact mode; AFM images are shown in Figure 1. Compared with the surface of the bare Ni surface (Figure 1a), high heating rates induce the partial dewetting of the Ni layer (Figure 1b), which displays a non-uniform thickness distribution, combining areas with hillocks and areas with voids apparently exposing the Mo underlayer. Ni films subjected to lower heating rates (Figure 1c) exhibit smoother surfaces with the presence of distant large pores. It is worth noting that micro-Raman spectroscopy measurements revealed that the quality of the graphene layer is roughly the same in the hillocks and the voids, suggesting that a thin Ni film is still covering the whole active area of the device.

**Figure 2:**
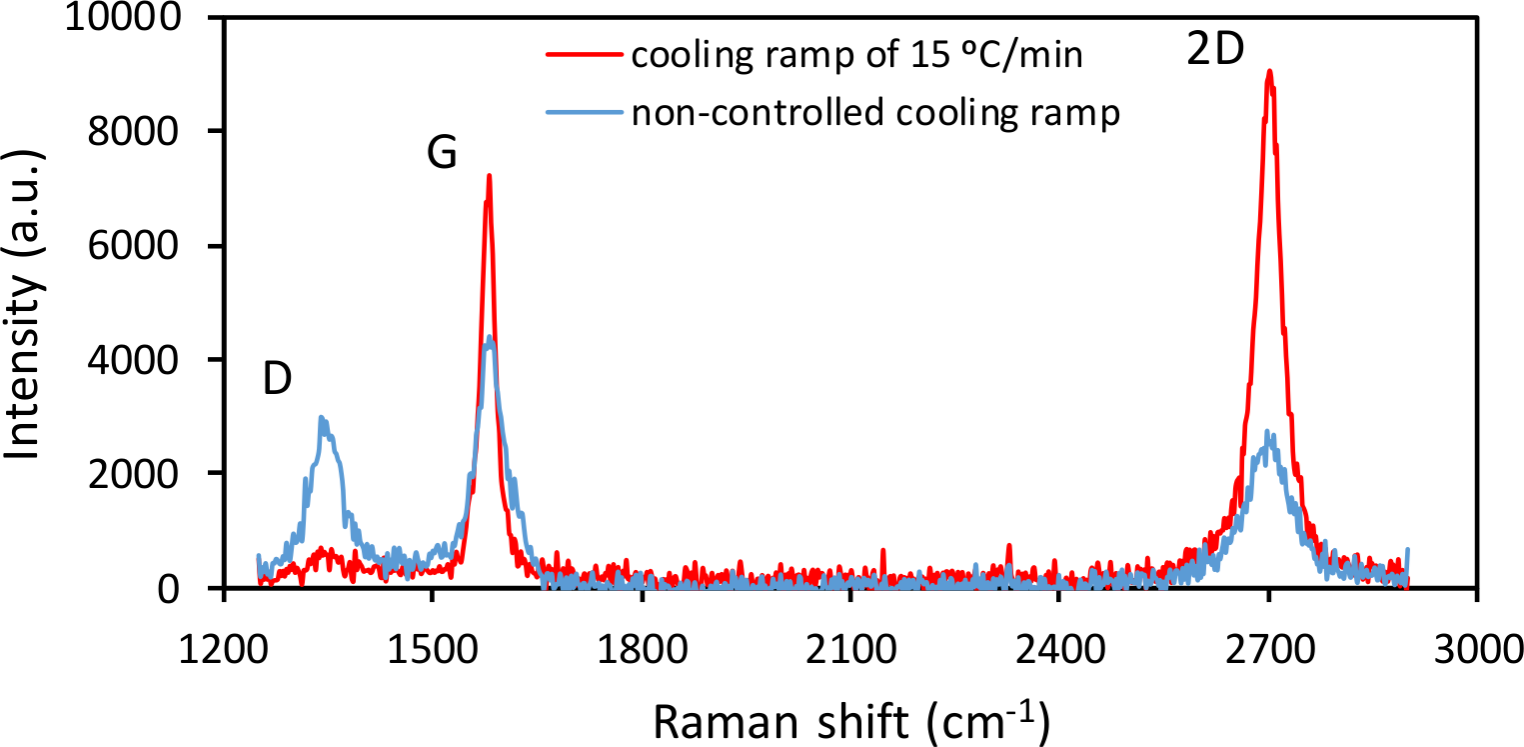
Typical Raman spectra of a graphene layer grown with an initial slow cooling rate of 15 °C/min and of a layer grown with non-controlled cooling rate. Note the low intensity of the defect band (D) in 1350 cm^−1^ for the sample grown with slow cooling rate.

Graphene layers were characterized by Raman spectroscopy with a B&BTek S415-532S spectrometer using a 532 nm laser operated at powers up to 50 mW. The Raman probe is mounted on a microscope that can focus the light to a spot of 2 µm in diameter on the sample surface. The uniformity and quality of the coverage was assessed by performing multiple measurements throughout the samples. Figure 2 shows typical Raman spectra of two graphene layers, one grown with a non-controlled cooling ramp and another grown with an initial cooling ramp of 15 °C/min. The FWHM of the 2D peak for this last sample is 40 cm^−1^, which suggests the presence of less than five weakly coupled layers [18]. Furthermore, the low-intensity D peak at 1350 cm^−1^ suggests that slowing down the cooling rate to reach 600 °C delivers graphene with better quality. The hydrophobic character of the surface was confirmed by dropping water on it with a pipette, which revealed the lack of liquid adhesion to the surface.

We also analysed the influence of the process parameters (pressure, Ar/C_2_H_2_ flow ratio and cooling rate) on the defect density and number of graphene layers. We noticed that both lowering the C_2_H_2_ flow rates and increasing the overall pressure (up to 12 mTorr) leads to a decrease of the D peak. We also observed that no graphene grows when the overall pressure raises above a given threshold (around 20 mTorr). A more detailed study of the influence of growth conditions on the characteristics of the graphene layers grown in our system can be found in [19].

**Figure 3:**
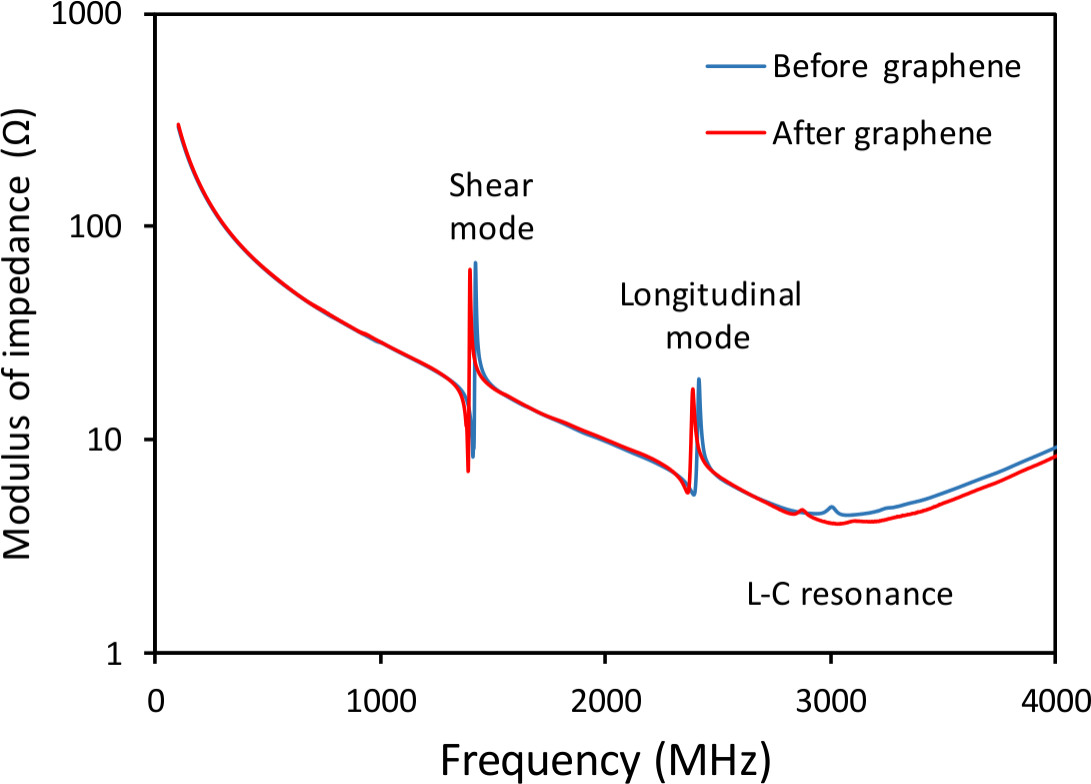
Electrical impedance spectra of the same device before and after graphene growth.

To check whether the performance of the SMRs was affected by the integration of graphene, we measured the electrical impedance spectra of the SMRs before and after the entire graphene integration process in the same device. The frequency response of a typical device, shown in Figure 3, suggests that the integration of graphene does not significantly degrade the performance of the SMRs; the resonant frequency is reduced by only 25 MHz while the coupling factor and quality factor of the resonators remain almost constant, with typical values of, respectively, 3% and 150 [20]. It is worth noting that the modulus of the impedance displays a minimum at around 3000 MHz due to the LC resonance that arises from the static capacitance of the resonator and the series inductance associated to the extension of the top electrode. To accurately track the resonant frequency during detection, these parasites can be removed by software. However, in practical oscillators this inductance should be removed (or minimized) by a careful design of the resonator and the fluidic system.

### Graphene functionalization

Two functionalization procedures of the graphene surface were explored. The first one is based on the conventional 2-step EDC/NHS protocol [13] to bind streptavidin covalently to the previously oxygen-plasma-treated surface, with the subsequent binding of biotinylated IgG antibodies. The second functionalization method is the direct non-covalent binding of streptavidin to the hydrophobic bare graphene surfaces (no plasma treatment is performed) to which the biotinylated IgG antibodies were bond. IgG antibodies were chosen as a typical antibody although any other biotinylated receptor for a given species could have been used. Both functionalization methods lead to a short distance between the antibody (target) and the resonator surface, thus maximizing the sensitivity [7]. Indeed, the closer the target to the surface the higher the interaction of the acoustic energy of the shear mode [8]. This is explained by the fact that the shear mode does not propagate to the liquid. There is only an evanescent acoustic field near the surface [21] that interacts with the added mass.

#### Covalent functionalization

The first step of this functionalization protocol is the incubation of the sample in a solution of 0.2 nM 1-ethyl-3-(3-dimethylaminopropyl)carbodiimide hydrochloride (EDC) with 0.8 mM *N*-hydroxysuccinimide (NHS) in 10 mM phosphate-buffered saline (PBS). This step requires the generation of carboxyl groups (–COOH) on the surface of the sample capable of binding the NH groups of the linker, in our case EDC.

**Figure 4:**
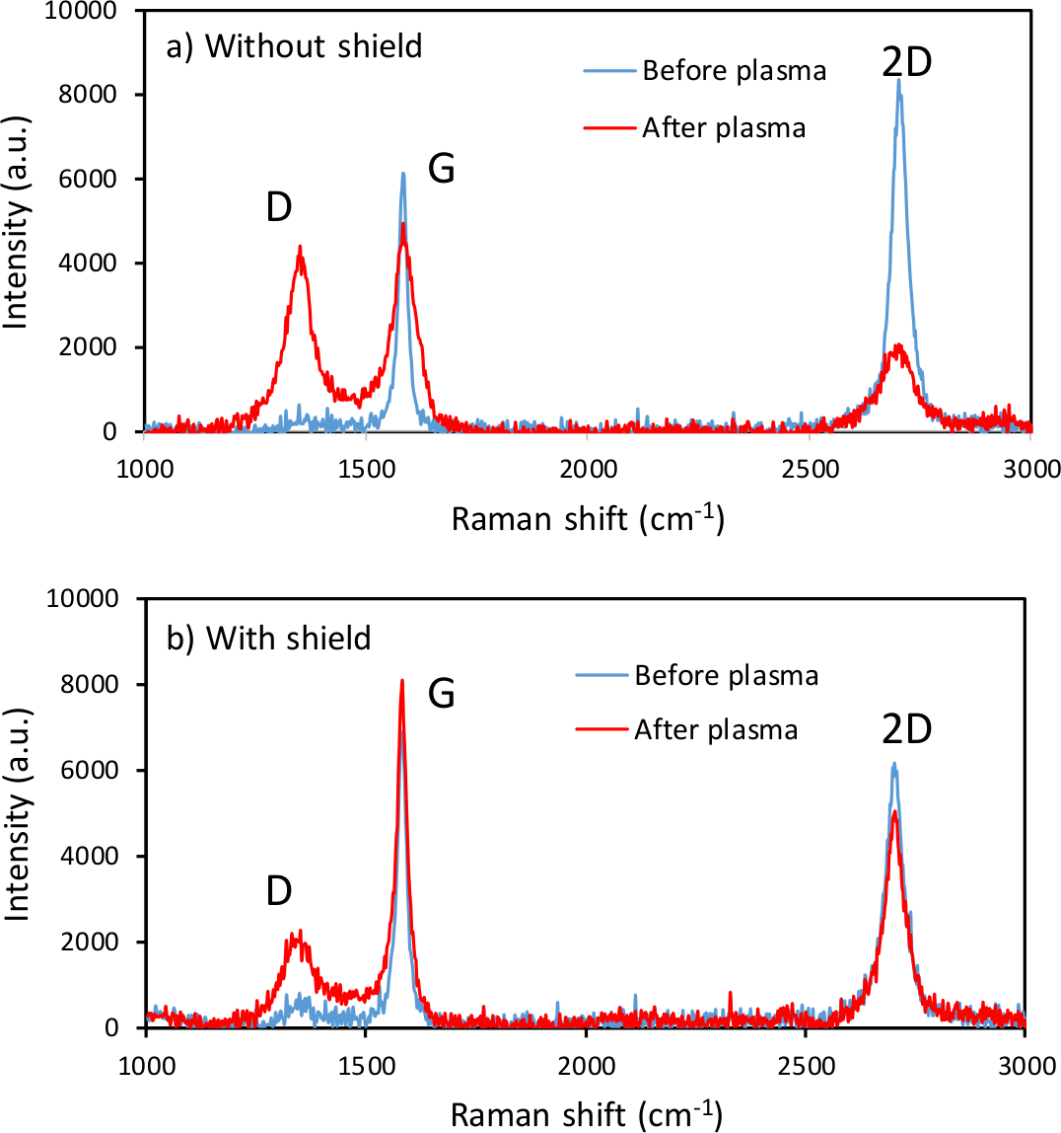
Raman spectra of samples of graphene grown on resonators before and after the O_2_ plasma treatment a) without and b) with glass shield. Note the degradation of the graphene layer when immersed in plasma without shield.

Since the as-grown graphene contained very low density of defects, being significantly hydrophobic, a specific treatment was required in order to promote the creation of controlled defects and the necessary carboxyl groups. This was achieved by subjecting the samples to a low-damage O_2_ RF-plasma in a reactive ion etcher. To prevent the negatively biased substrates from being bombarded by the energetic ions present in the plasma, a glass shield was placed 1 mm apart from the surface of the substrates. This way, only thermalized reactive oxygen radicals and neutral reactive species were able to reach the graphene surface by efficient diffusion through the gas discharge [22]. The process parameters were previously optimized (50 W, 25 mTorr and 25 sccm O_2_ flow for our system). The samples were characterized by Raman spectroscopy before and after each plasma treatment (of different durations) to monitor the amount of defects and the integrity of the graphene layer. Figure 4 highlights the effect of the plasma treatment in unshielded and shielded samples. The Raman spectra reveal that the number of defects increases in both samples, as evidenced by the rise in the D peak. The increase of this peak can be attributed to the appearance of COOH groups [23]. However, the intensities of the G and 2D peaks are barely affected by the plasma treatment in shielded samples, indicating that the structural integrity of the graphene layer is preserved. Thus, we can conclude that the O_2_ plasma treatment of adequately shielded graphene layers successfully promotes the creation of defects without significant damage of the graphene structure. The evidence that these defects are mainly carboxyl groups is the efficient binding of amine groups of the EDC linker, evinced by the detection measurements that will be show in this section.

**Figure 5:**
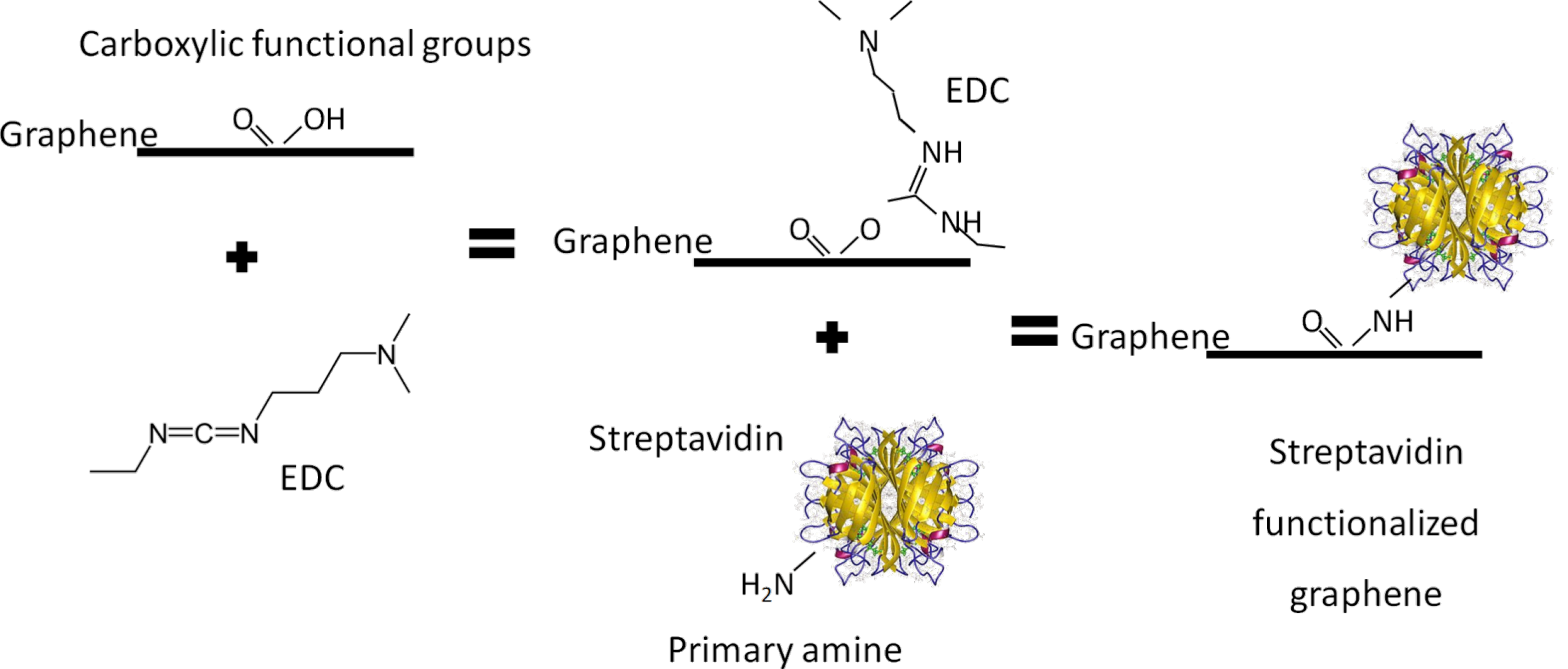
Schematic representation of the EDC covalent functionalization of graphene with streptavidin.

An additional effect of the O_2_ plasma treatment is that the initially hydrophobic graphene layer becomes hydrophilic, as observed by analysing the adhesion of a droplet of DI water to the surface; the adhesion improved after the plasma treatment, indicating the increase of hydrophilicity. This is important as this treatment strongly determines the type of binding of the reagents to the surface in covalent and non-covalent bio-functionalization. Figure 5 shows a schema of the covalent functionalization process.

**Figure 6:**
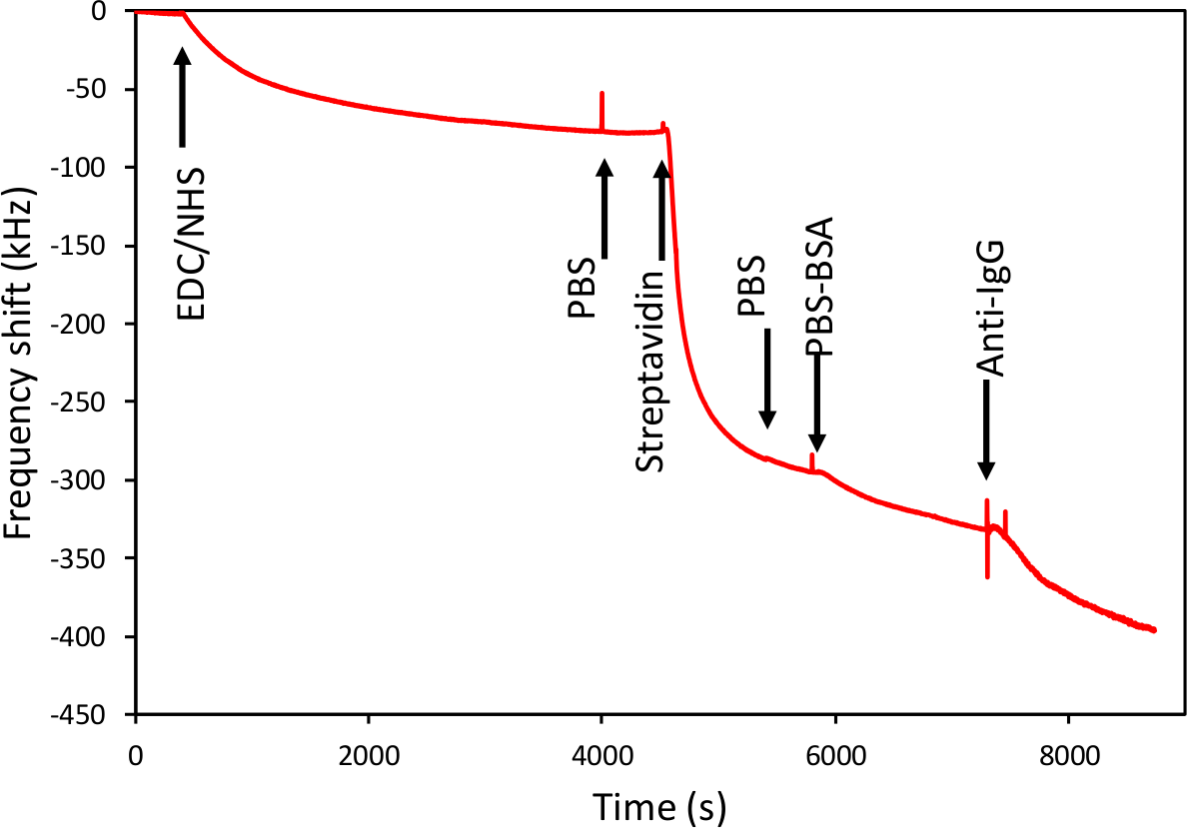
Resonant frequency evolution of a resonator during the covalent functionalization and anti-IgG antibody detection process. The arrows indicate the moments at what the liquids are changed.

After the plasma treatment, the samples were exposed to the different solutions by circulating the liquids containing the reagents with the aid of the peristaltic pump while the frequency response of the resonator was continuously recorded. Figure 6 shows the time evolution of the resonant frequency of a resonator subjected to the above mentioned process; the arrows indicate the times at which the different reagents are introduced. The samples were first incubated in the EDC/NHS solution for 1 h at room temperature. After a PBS washing, a 5 µg/mL concentrated streptavidin solution was circulated through the system, which produced a strong frequency shift due to the covalent binding of streptavidin to the EDC on the device surface. Then BSA 0.1% in weight in PBS was used as blocking agent and, finally, the surface was exposed to biotinylated anti-IgG antibodies by circulating a 330 nM solution in the system. It is worth mentioning that during the surface blocking with PBS, the signal did not reach the saturation. This behaviour was observed in most of the experiments we carried out both during covalent and non-covalent functionalization. We hypothesize that PBS diluted in BSA attach gradually to all the surface sites not having bound streptavidin during the first stages of the exposure. However, once the surface is fully covered, BSA might start to bond to the already occupied sites, which would be in agreement with the observed linear drop of the resonant frequency as a function of time. In addition, slight temperature variations could also account for the observed trend.

The response of the SMR-based gravimetric sensor is comparable to that obtained in similar biosensors subjected to a silane-based functionalization [24].

#### Non-covalent functionalization

According to previous studies [25], non-covalent binding is possible on pristine hydrophobic graphene, which prompted us to investigate the direct non-covalent binding of streptavidin to our bare graphene hydrophobic surfaces. According to [13], streptavidin binds to the sidewalls of carbon nanotubes (CNTs) by means of hydrophobic interactions. It was expected it would bind also to graphene, which is comparable to unfolded CNTs. This significantly simplified the functionalization process, since the EDC/NHS incubation of the plasma-treated graphene was omitted.

**Figure 7:**
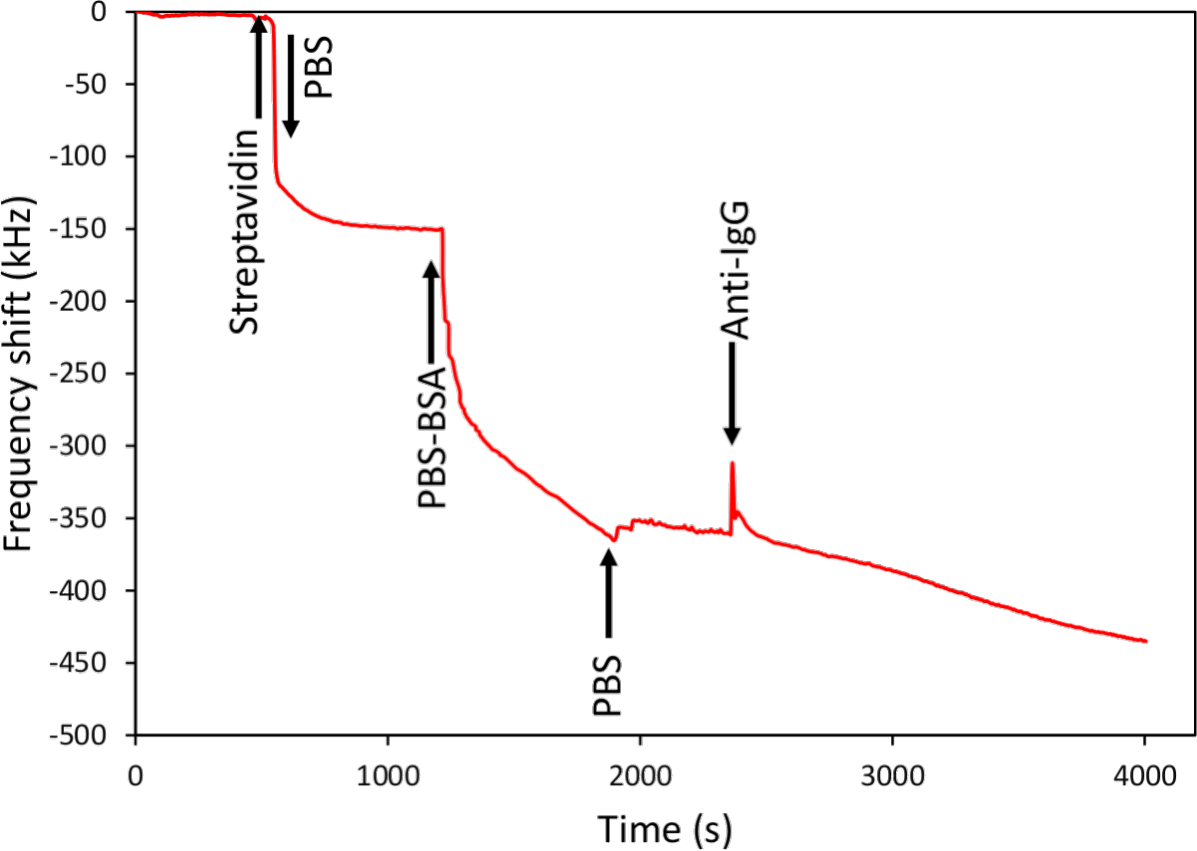
Resonant frequency evolution of a resonator during the non-covalent functionalization with anti-IgG antibody. The arrows indicate the moments at what the liquids are changed.

In the present experiment (Figure 7), after the graphene growth, the samples were mounted in the peristaltic pump system and washed with PBS 10 mM until the resonant frequency was stabilised. Figure 7 shows the time evolution of the resonant frequency of a resonator during the non-covalent process; again the arrows point out the times at which the different liquids are introduced in the fluidic system. In this approach, a solution of streptavidin 5 µg/mL in 10 mM PBS was fed to the system. Previous tests revealed that saturating the surface with streptavidin resulted in less efficient subsequent steps, because streptavidin can form multilayers stacks leaving fewer sites for antibody binding. Having this in mind, in this case the flow of streptavidin buffered in PBS was interrupted after a frequency drop of around 100 kHz, to prevent the surface from saturating. This resulting frequency drop revealed that streptavidin was being bound to the surface and the fact that the frequency did not increase again when the flow was interrupted indicated that the binding was stable and permanent. After frequency stabilization, a solution of 1 µg/mL of BSA in 10 mM PBS buffer was circulated until the frequency stabilized again. This step prevented from unspecific binding on the surface as BSA completely covered all the non-streptavidin bound surface. After washing with PBS, the biotinylated anti-IgG antibody 330 nm was fed to the system. In this step the frequency shift of 110 kHz indicates the binding of anti-IgG to the surface. This frequency shift is slightly higher than for the covalent functionalization case although such in that case it does not saturate the surface.

The two functionalization methods appear to be remarkably similar. However, although non-covalent bonding seems much simpler and apparently yields similar results than the covalent functionalization protocol, some considerations should be taken into account when it comes to choose the most appropriate. Firstly, one has to consider the lack of specificity of the non-covalent bond. Given that streptavidin directly binds to graphene, other species with similar functional groups (primary amine) may also do so. Therefore, high-purity reagents are needed for a proper functionalization. Additionally, one has to ensure the effective blocking of the non-covered surface to avoid false positives. Plasma-treated surfaces for covalent binding seem to be less reactive and then more selective for binding the desired species. A deep analysis of the advantages and drawbacks of the non-covalent functionalization method, which is beyond the purpose of this work, needs to be done before concluding that this functionalization method is as reliable as the covalent one. However, regardless of the functionalization method, graphene directly grown on gravimetric sensors emerges as a promising platform for the bio-functionalization of industrially scalable biosensors.

## Conclusion

AlN-based solidly mounted resonators (SMRs) operating in the shear mode were covered with graphene layers intended as functionalization platforms for gravimetric biological sensors. The graphene layers were directly deposited on the resonators through a relatively low-temperature LPCVD process that allowed for the preservation of the integrity of the whole multi-layered structure as well as its electrical response. The graphene-covered resonators were tested as biosensors for the detection of anti-IgG antibodies. Two functionalization protocols were explored. In the first one, EDC/NHS chemistry was used to covalently bind streptavidin molecules to the surface of the sensors after a low-damage O_2_ radical plasma treatment inducing a controlled density of defects on the graphene layer. In the second approach, non-covalent functionalization of pristine as-grown graphene was also demonstrated by firmly binding streptavidin to untreated graphene surfaces. Although both functionalization approaches appear to provide similar results, their suitability should be analysed in terms of their specificity to particular targets.

## Experimental

### Fabrication of resonators

**Figure 8:**
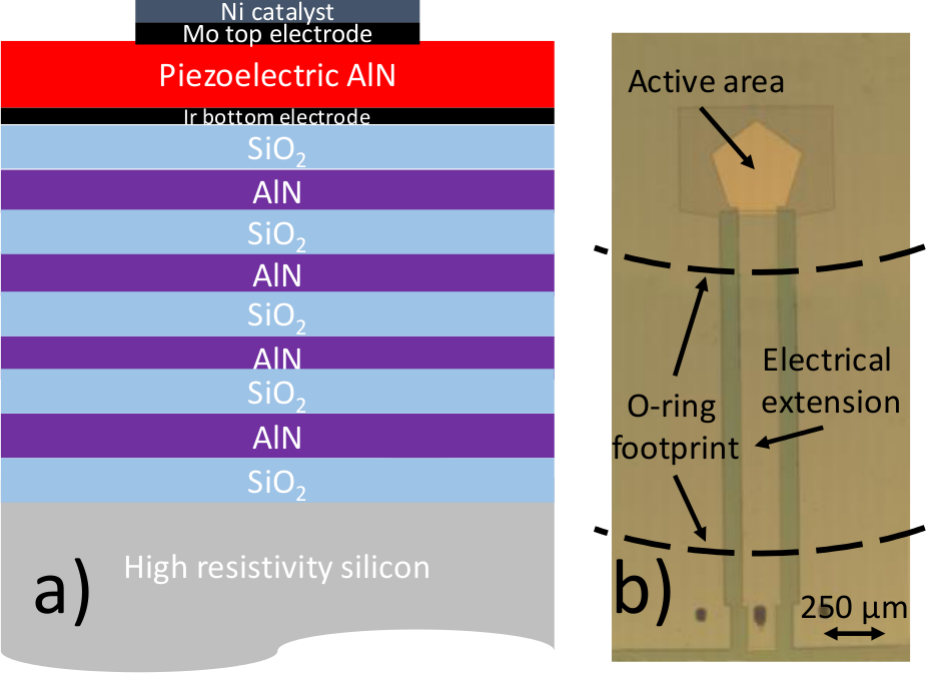
a) Schematic cross section of a typical AlN-based SMR showing the Ir/AlN/Mo/Ni piezoelectric stack and the acoustic reflector and b) optical picture of a device showing the electrical extensions and the footprint of the fluidic O-ring on the device.

The performance of the resonators was first simulated using Mason’s model in order to adjust the thicknesses of the various films that compose the whole device, which includes the silicon substrate, the layers with high (AlN) and low (SiO_2_) acoustic impedance of the fully insulating reflector, the Ir/AlN/Mo piezoelectric stack and the Ni catalyst that covers the active area of the device (Figure 8a). All these layers were adjusted to set the resonant frequency to the desired value and to achieve the best performance of the SMRs in terms of quality and electromechanical coupling factors. To fit the fluidic system, the electrical contacts to the resonators were set apart from the active area of the devices, which entailed extending the electrical pads (see Figure 8b where the O-ring sealing footprint is sketched) [26].

All the films involved in the SMR structure were deposited by sputtering, except the Ir bottom electrode and the Ni catalyst that were e-beam evaporated. The deposition conditions of the AlN and SiO_2_ layers in the reflector were carefully adjusted to minimize residual stresses in each film. The final devices were capable of sustaining temperatures as high as 1000 °C for at least two hours, without significant degradation of their performance [27]. Although the devices could withstand such extreme conditions, the growth of graphene was carried out at lower temperatures to prevent the devices from degradation, no matter how small.

To excite shear modes with mass displacement parallel to the surface–liquid interface, more appropriate for in-liquid operation than longitudinal modes, the AlN active films were purposely grown with the *c*-axis uniformly tilted with respect to the surface normal. Details of AlN deposition and performance of the shear-mode resonators can be found in [20].

### Growth of graphene

**Figure 9:**
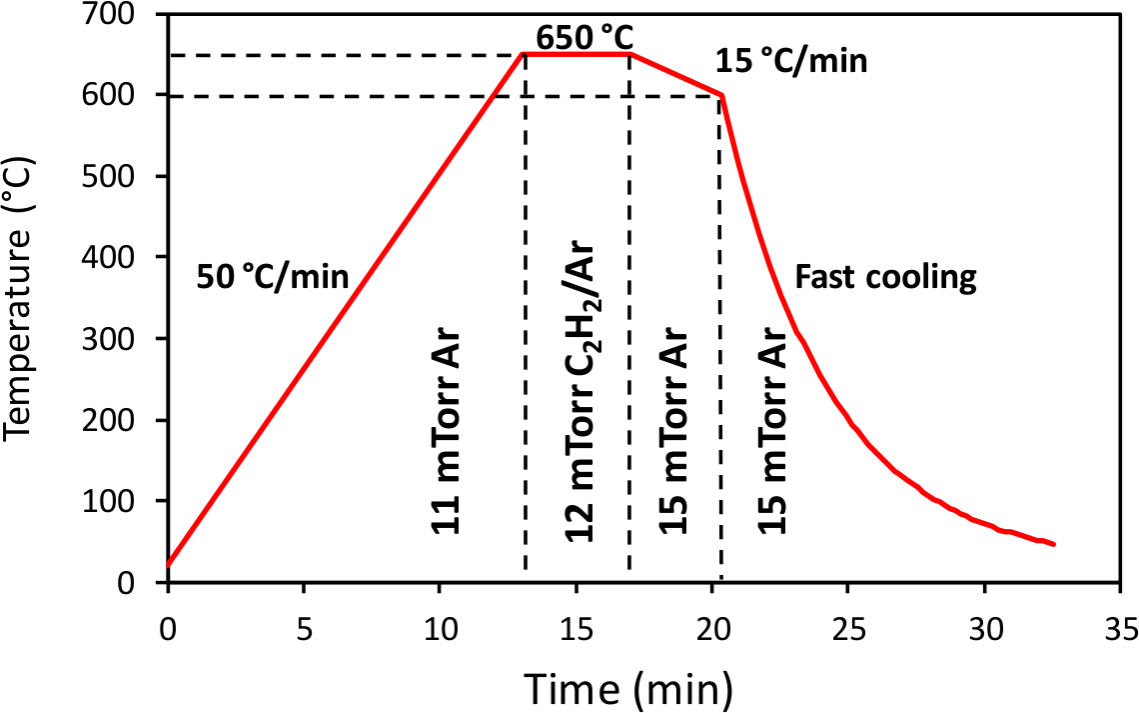
Temperature evolution during the graphene growth process. Four stages are represented, corresponding to the sample heating in pure Ar atmosphere, the C_2_H_2_ supply (diluted in Ar), the slow cooling in pure Ar atmosphere, and the fast cooling, also in Ar atmosphere.

Graphene was grown on top of the SMRs in a custom-built, cold-wall low-pressure CVD reactor. Although this kind of CVD process is frequently used for graphene growth [28], as far as we know it has not been used previously for the direct growth of graphene on functional devices. Before starting the CVD process the Mo top electrode defining the active area of the resonators was covered with a thermally evaporated 100 nm thick Ni films that acted as catalyst for the decomposition of acetylene (C_2_H_2_), the source of the carbon atoms. The samples were then introduced in the CVD chamber, which was initially evacuated to a base pressure in the range of 10^−7^ Torr before being filled with Ar at a pressure of 11 mTorr to start the thermal sequence for graphene growth shown in Figure 9.

In a first step, the samples were heated to 650 °C at a rate of 50 °C/min, during which the Ni film was cleaned from oxides and impurities. Afterwards, acetylene was fed into the chamber while adjusting the C_2_H_2_/Ar mixture to keep the pressure constant at 12 mTorr. The samples were exposed to this ambient for 5 min, during which C_2_H_2_ was catalytically decomposed on the Ni surface letting the resulting carbon atoms to diffuse into the Ni film. In the next step the C_2_H_2_ flow was cut off to allow the samples to cool down to 600 °C at a slower controlled rate of 15 °C/min under an Ar pressure of 15 mTorr. During this stage, carbon atoms segregated from the Ni film to form the graphene layer on its surface. The cooling rate was critical because the graphene film maintains a quasi-equilibrium with the atmosphere and the carbon inside the Ni film. The number of layers of the resulting graphene depended on the cooling rate and time of this step. Finally, the heater was switched off to let the sample freely cool down to room temperature keeping the Ar pressure at 15 mTorr. Since the graphene layer stabilizes when the temperature is low enough, the cooling should be as quick as possible. Under our experimental conditions the temperature reaches 400 °C in less than 2 min, with an initial cooling rate close to 200 °C/min.

### Bio-detection system

The sensors were characterized by measuring the electrical impedance of the resonators from 100 MHz to 6 GHz with an Agilent N5230A network analyser using calibrated RF probes for on-wafer contacting. In order to verify whether the high-temperature process affected the performance of the devices, the SMRs were carefully assessed before and after the growth of the graphene layer.

**Figure 10:**
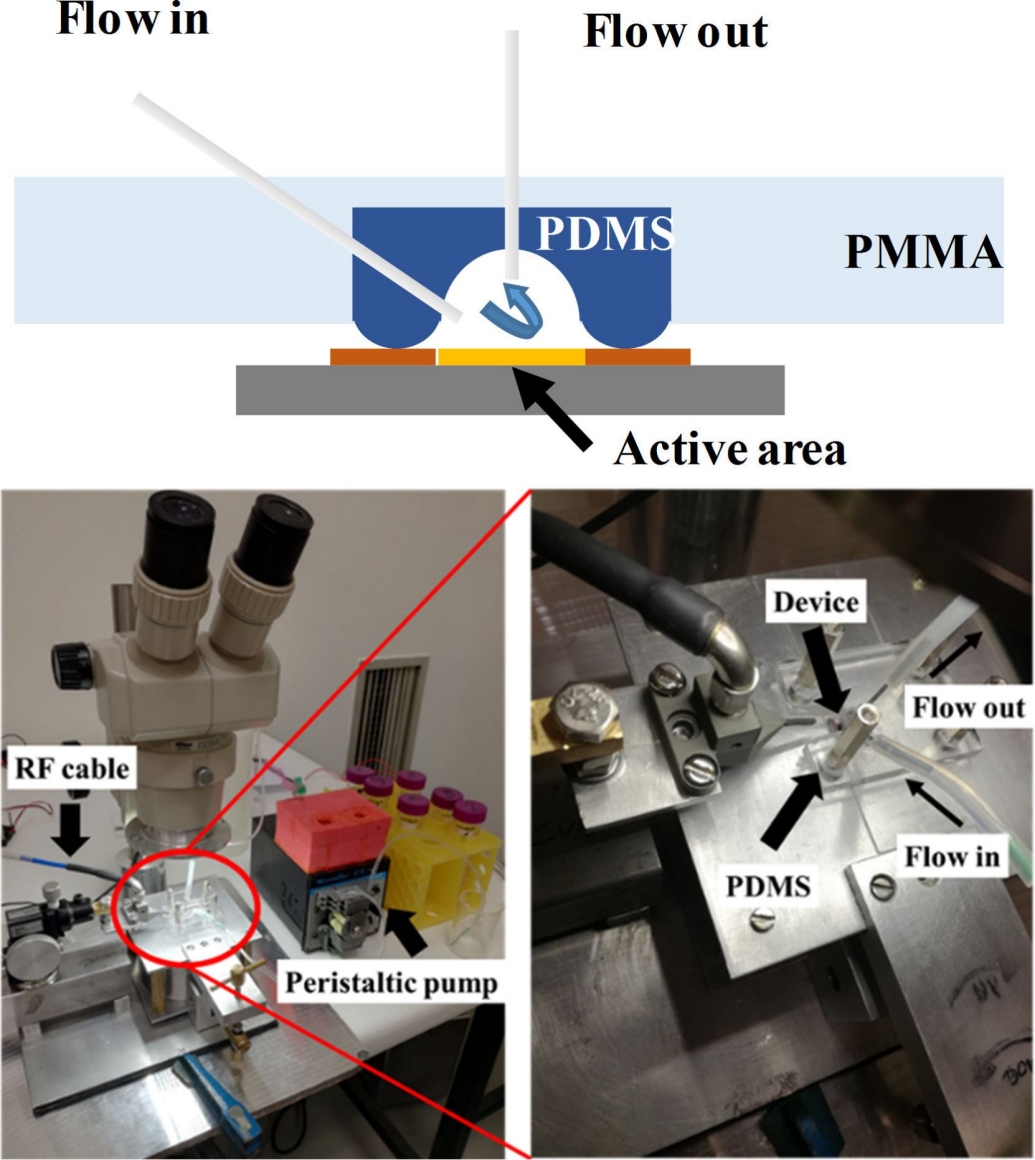
Experimental setup for bio-detection: sketch of the fluidic system and photos of the experimental arrangement.

For in-liquid measurements, we used a fluidic system made of PMMA that includes a PDMS chamber with an O-ring to seal a 30 µL cavity around the active area of the device (see Figure 10). A peristaltic pump feeds the liquid into the cavity at a rate of 100 µL/min. The liquids can freely flow for washing purposes or be recirculated when the incubation times are long. The whole volume of the circuit is 400 µL. To accurately track the resonant frequency during the bio-detection process, the real part of the admittance was fitted to a rational function in a narrow frequency interval around its maximum at the resonant frequency. The location of the zeros of its first derivative was calculated and the resonant frequency identified. This process was controlled with a LabVIEW^®^ application that allowed assessing the frequency with less than 1 kHz accuracy each 7 s. Figure 10 shows the experimental setup.
